# Functional role of UNC13D in immune diseases and its therapeutic applications

**DOI:** 10.3389/fimmu.2024.1460882

**Published:** 2024-10-14

**Authors:** Van-Thanh Duong, Dongjun Lee, Yun Hak Kim, Sae-Ock Oh

**Affiliations:** ^1^ Department of Anatomy, School of Medicine, Pusan National University, Yangsan, Republic of Korea; ^2^ Department of Convergence Medicine, School of Medicine, Pusan National University, Yangsan, Republic of Korea; ^3^ Department of Biomedical Informatics, School of Medicine, Pusan National University, Yangsan, Republic of Korea

**Keywords:** UNC13D, cytotoxic granule secretion, immune diseases, FHL3, gene therapy

## Abstract

UNC13 family (also known as Munc13) proteins are evolutionarily conserved proteins involved in the rapid and regulated secretion of vesicles, including synaptic vesicles and cytotoxic granules. Fast and regulated secretion at the neuronal and immunological synapses requires multiple steps, from the biogenesis of vesicles to membrane fusion, and a complex array of proteins for each step. Defects at these steps can lead to various genetic disorders. Recent studies have shown multiple roles of UNC13D in the secretion of cytotoxic granules by immune cells. Here, the molecular structure and detailed roles of UNC13D in the biogenesis, tethering, and priming of cytotoxic vesicles and in endoplasmic reticulum are summarized. Moreover, its association with immune diseases, including familial hemophagocytic lymphohistiocytosis type 3, macrophage activation syndrome, juvenile idiopathic arthritis, and autoimmune lymphoproliferative syndrome, is reviewed. Finally, the therapeutic application of CRISPR/Cas9-based gene therapy for genetic diseases is introduced.

## 1 Introduction

Fast and regulated secretion of vesicles is critical for neurotransmission at the neuronal synapse and for defense at the immunological synapse (1–4). This requires multiple steps, from the biogenesis of vesicles to membrane fusion, and a complex array of proteins for each step. The UNC13 family (also known as Munc13) proteins are evolutionarily conserved proteins responsible for rapid and regulated secretion of vesicles, including synaptic vesicles and cytotoxic granules (5–8). Their expression and roles in various tissues, including the nervous and immune systems, have been documented previously (5, 6, 9, 10). In addition, their pathological roles have been observed in genetic diseases, such as familial hemophagocytic lymphohistiocytosis, and various types of cancers (11–13). UNC13 family proteins have been observed in primitive animals, even before the emergence of neurons (14, 15). Four isoforms of the UNC13 family have been identified in mammals; these include UNC13A, UNC13B, UNC13C, and UNC13D (16). UNC13A and UNC13C are specifically expressed in nervous tissues. *UNC13B* is transcribed into two principal isoforms via alternative splicing—*ubUNC13B* is ubiquitously expressed, whereas *bUNC13B* is restricted to the brain (9, 17). Northern blotting and quantitative reverse transcription-PCR analysis revealed that UNC13D expression is widespread, with strong expression in hematopoietic cells, including CD4 and CD8 T lymphocytes, CD19 B lymphocytes, natural killer (NK) cells, and CD14 monocytes. Regarding non-hematopoietic cells, UNC13D exhibits strong signals in the lungs, liver, pancreas, and placenta, whereas only modest signals are observed in the brain, heart, skeletal muscle, and kidney (6). A broad review of the UNC13 family members has been published previously (5, 16). The present study focuses on recent progress in research on the role of UNC13D in the immune system.

## 2 Structural characteristics of UNC13D

**Figure 1:**
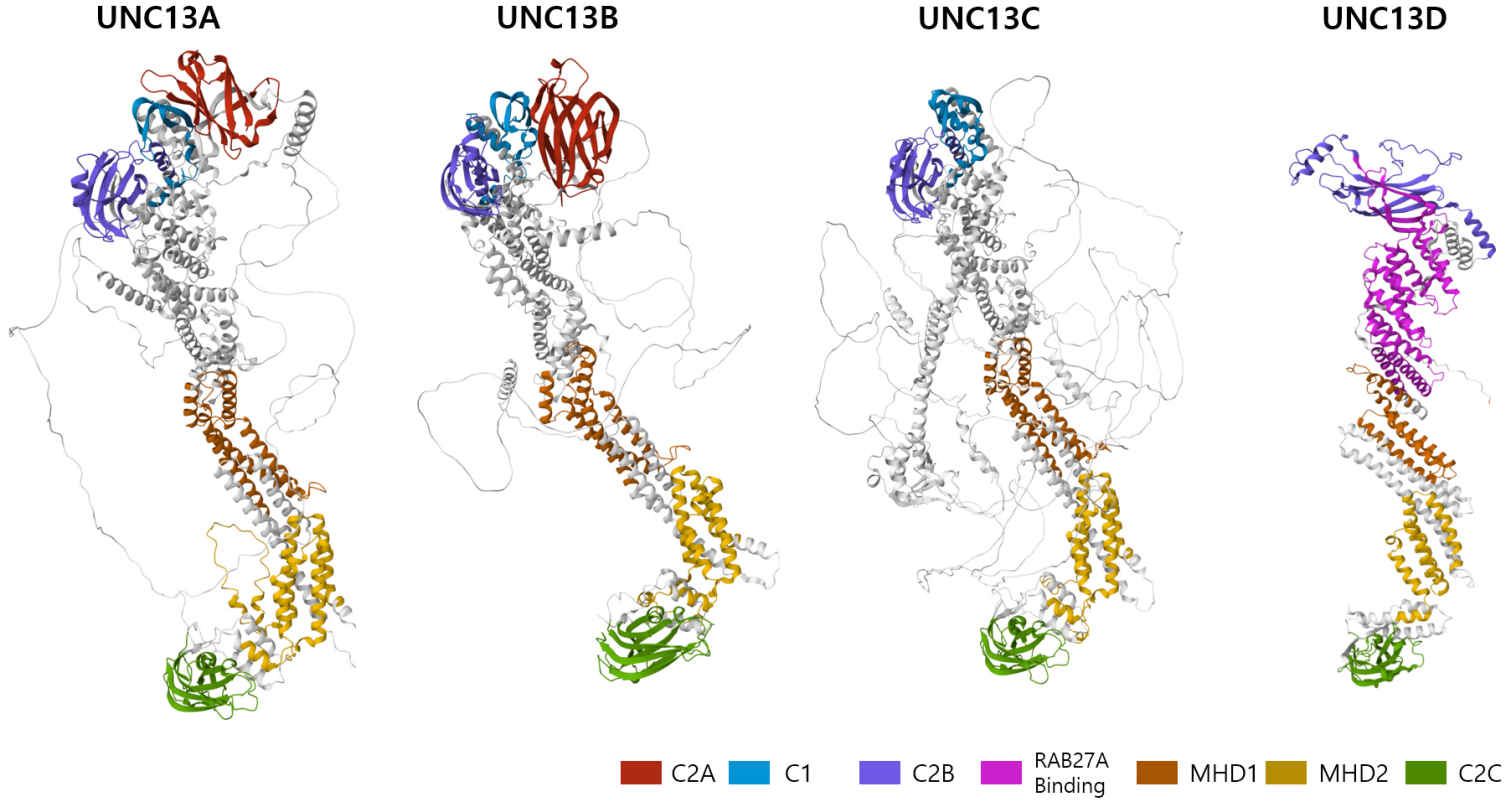
3D structure of the human UNC13 family members predicted by AlphaFold (https://alphafoldserver.com/). Critical domains are indicated by color code.

Structural characteristics of the UNC13 family members have been described previously (8, 9). Although the core domains of UNC13 members are conserved in UNC13D and brain-specific angiogenesis inhibitor 1 associated protein 3, they exhibit structural differences from the cognate UNC13 proteins because they lack three specific domains—an N-terminal C2 domain (C2A), a calmodulin-binding domain, and a diacylglycerol-binding C1 domain (**Figure 1**). The calmodulin-binding domain is located between the C2A and C1 domains (not marked in **Figure 1** for simplicity). In addition to these three characteristic differences, UNC13D has fewer disordered regions than the other family members, which suggests that it is more rigid than other members (**Figure 1**).

Structurally, UNC13D contains two C2 domains separated by long sequences that contain two regions known as Munc13-homology domains (MHD1 and MHD2) and RAB27A binding domain (**Figure 1**) (8). These domains display distinctive topologies based on the circular permutation of their β strands, wherein the N- and C-termini remain either at the top or bottom of the C2-β sandwich. Type I topology is found in the first C2 domain (C2A), which is comparable to cPLA2 and PLCδ1. Type II topology is found in the second C2 domain (C2B), similar to that encountered in the C2A and C2B domains of synaptotagmin 1 as well as in rabphilin (18, 19). In addition, the MHD-containing region, which separates the two C2 domains, is entirely made up of α helices, some of which have specific lengths and distinct heptad repeats, indicating structures generating coils. UNC13D and its partners may interact through this hypothesized coil-forming structure (6). Furthermore, amino acids 240–543, which constitute the C-terminal end of the C2B domain, are necessary for binding to Rab27a (**Figure 1**). In particular, residue 280FQLIHK285 is crucial for binding (20).

Unlike other UNC13 proteins, UNC13D does not have the diacylglycerol-binding C1 domain that is essential for neuronal priming. However, the two C2 domains shared by UNC13D and all other UNC13 homologues have been implicated in vesicle priming and exocytosis (8). UNC13D is a versatile protein that associates with numerous molecules and performs diverse functions. Apart from Rab27a, a well-known interacting partner of UNC13D, other Rab GTPases, such as Rab7, Rab11, and Rab37, interact for vesicle trafficking. Several studies have reported that UNC13D is associated with Cd63, Stk24, Ccm3, Doc2 alpha, and S100A10, which regulate exocytosis (**Table 1**).

**Table 1:** UNC13D and its binding proteins.

Interacting protein	Function	Interacting region	References
Rab27a	Tethering, exocytosis	240-543	(20–22)
Rab11	Vesicle trafficking, exocytosis	Not determined	(13, 23, 24)
Cd63	Exocytosis	1–899	(13, 25)
Stk24	Exocytosis	C2C	(26)
Ccm3	Exocytosis	C2B	(26)
Doc2a	Exocytosis	1–89, 215–400	(27)
Rab37	Mast cell degranulation	540-914, 239-289	(28)
S100a10	Exocytosis	C2B, C2C	(29)
Stx7	Late endosomal maturation	Not determined	(30)
Vamp8	Late endosomal maturation	Not determined	(30)
Rab7	Exocytosis	Not determined	(31)
Stx2	Human neutrophil elastase stimulation	Not determined	(32)
Rhog	Docking, fusion	Not determined	(33)
Sting1	Negative regulator of the STING-mediated innate immune response.	C2B, MHD1/2	(34)

## 3 Role of UNC13D in immune cells

### 3.1 Role of UNC13D in the biogenesis of cytotoxic granules

**Figure 2:**
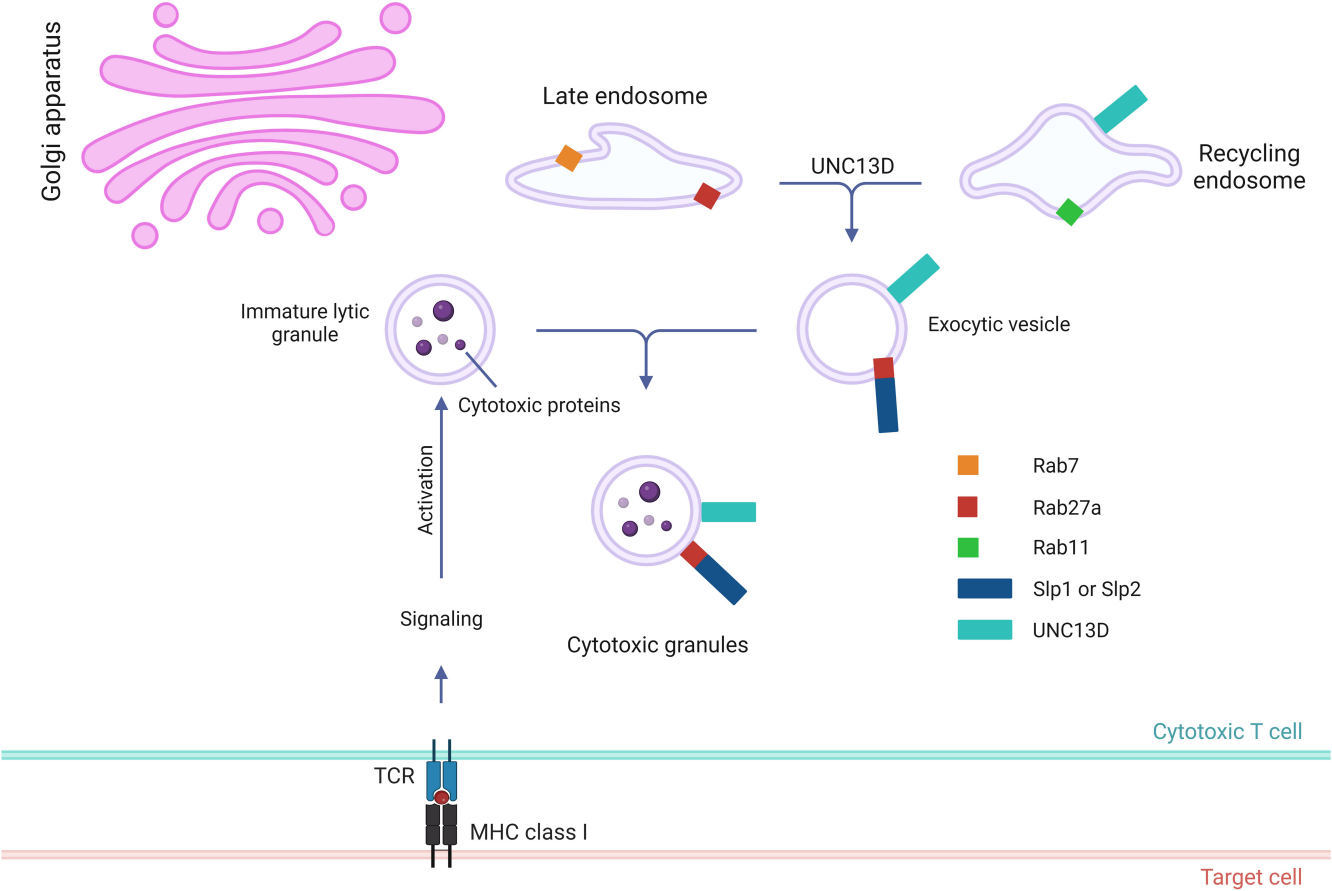
Role of UNC13D in the biogenesis of cytotoxic granules. Stepwise genesis of cytotoxic granules from late and recycling endosomes via exocytic vesicle is presented. Cytotoxic proteins, such as granzymes, perforin, and granulysin, are synthesized in the endoplasmic reticulum before being transported to the Golgi apparatus. UNC13D is responsible for coordinating the subsequent interaction between recycling endosomes via Rab11 and late endosomes via Rab7 to generate exocytic vesicles that are specific to the regulated secretory pathway. These vesicles contain effector proteins essential for exocytosis, such as UNC13D, Rab27a, and synaptotagmin-like proteins (Slps).

To destroy the target cells, immune cells (e.g., NK and CD8 T cells) secrete cytotoxic granules into them (7). The multistep generation of cytotoxic granules containing perforin, granzymes, and granulysin has been documented using electron microscopy and kinetic tracer uptake experiments (35–37). This cytotoxic process is triggered by the recognition of target cells via immune receptors and plasma membrane (PM) proteins (38). Cytotoxic T lymphocytes (CTLs) are activated through the interaction of T-cell receptors (TCR) with MHC class I molecules and the recognition of antigenic peptides derived from the target cells. NK cell cytotoxicity is initiated by the ligation of activating receptors and is regulated by the recognition of self MHC class I molecules. Cytotoxic lymphocytes employ two distinct pathways to perform their effector functions. The first step involves the binding of the Fas ligand on CTLs and NK cells to its receptor on the target cell, leading to oligomerization of the receptor and apoptosis of the target cells (39). The cytotoxic lytic granule pathway, also known as the second pathway, is a rapid and efficient exocytic transport pathway for vesicular structures that carry lytic molecules such as perforin and granzymes (4). The early stage in cytotoxic granule generation involves merging of lysosomes with ‘exocytic vesicles’ (**Figure 2**) (20, 24, 40). In cytotoxic T cells, the exocytic vesicles originate from the fusion of late endosomes, containing Rab27a and Rab7, with recycling endosomes, containing Rab11 and UNC13D. During the fusion of the late and recycling endosomes, UNC13D, localized in the recycling endosome, plays a critical role by interacting with Rab27a, localized in the late endosome. Similarly, in NK cells, CD16, NKG2D, and 2B4 recruit UNC13D to the cytotoxic granules (41). In addition, UNC13D localizes to Rab7+ and Rab27b+ vesicles and merges with Rab11+ endosomes to generate UNC13D+/Rab7+/Rab11+ endosomal vesicles in mast cells (31). Nevertheless, the mechanism underlying UNC13D localization needs to be further explored.

### 3.2 Role of UNC13D in the tethering and docking of cytotoxic granules

**Figure 3:**
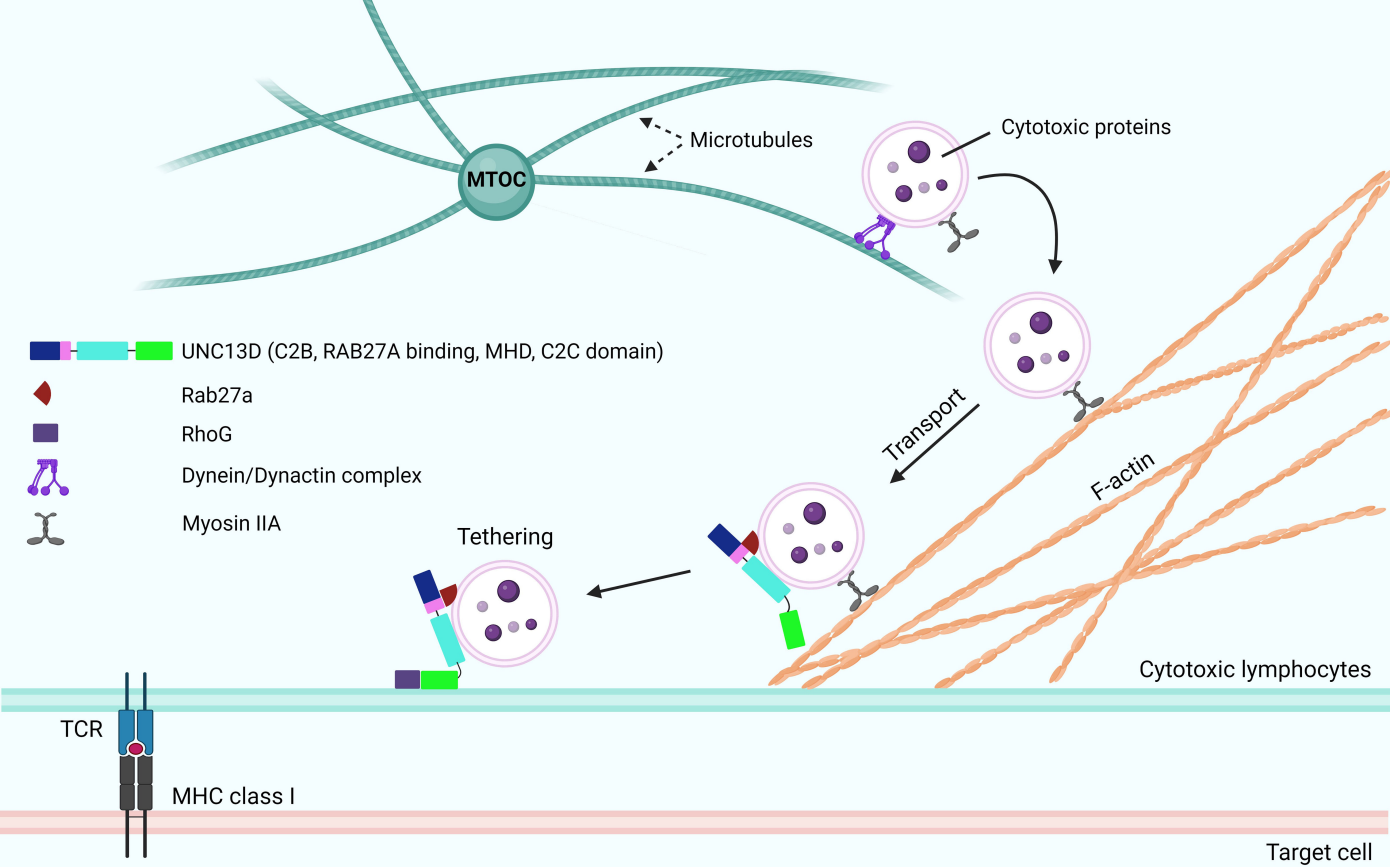
Role of UNC13D in the tethering and docking of cytotoxic granules. Following the activation of receptors of cytotoxic lymphocytes and initiation of signaling cascades, the cytotoxic granules are transported toward the MTOC along microtubules in a dynein-dynactin complex-dependent manner. The MTOC and granules polarize toward the contact area of the target cell, where the granules switch from microtubules to an F-actin network, at the immunological synapse. Subsequently, they move through the cortical filamentous actin meshwork because of the activity of the actin motor protein myosin IIA. Through the involvement of Rab27a and RhoG, the cytotoxic granules can transport and attach to the plasma membrane. While UNC13D interacts with Rab27a and associates with lytic granules through its MHD domains, the tethering of docked granules to the plasma membrane is orchestrated by UNC13D, which interacts with RhoG through its phospholipid-binding C2C domain. MTOC, microtubule organizing center; F-actin, filamentous actin; MHDs, Munc-homology domains; RhoG, Ras Homolog Family Member G.

After the generation of cytotoxic granules, which are meant to fuse with the PM, they need to be transported around the immunological synapse to create a readily releasable pool for the secretion (**Figure 3**). To maintain this pool, the cytotoxic granules must be loosely docked (tethered) to increase their dwelling time (42) near the PM. During this tethering step, prior to tight docking by soluble NSF attachment protein receptor (SNARE) proteins, Rab27a proteins and their effector molecules play critical roles (43). The interaction between Rab27a and Slp2a, a synaptotagmin-like protein, is critical for localizing cytotoxic granules near the PM via C2 domains (44, 45). The finding that UNC13D has C2 domains that can associate with PM phospholipids and interact with Rab27a suggests that UNC13D regulates the tethering of cytotoxic granules to the PM. The UNC13D mutant, which cannot bind to Rab27a, severely impairs the stalling behavior upon the initiation of immune receptor signaling (20). In addition, UNC13D restricts cytotoxic granules to the PM after the lipopolysaccharide stimulation in neutrophils (46). Because the binding of C2 domains of UNC13D to PM phospholipids requires Ca^2+^ (47) and no significant defects are observed in the tethering step in patients with familial hemophagocytic lymphohistiocytosis type 3 (FHL3) having UNC13D mutations (6), further studies are required to elucidate the role of UNC13D in the tethering step.

Recently, Kalinichenko et al. identified RhoG as a new binding partner of UNC13D (33). They reported that the protein-protein interaction between RhoG and UNC13D is crucial for docking UNC13D to the PM, as it enables membrane fusion. Mechanistically, UNC13D lacks a C1 lipid-binding domain and cannot bind to lipids alone. In contrast, RhoG can bind to phospholipids in an activation-dependent manner; thus, the assistance of RhoG is required to bind to membrane lipids.

### 3.3 Role of UNC13D in the priming and docking of cytotoxic granules

**Figure 4:**
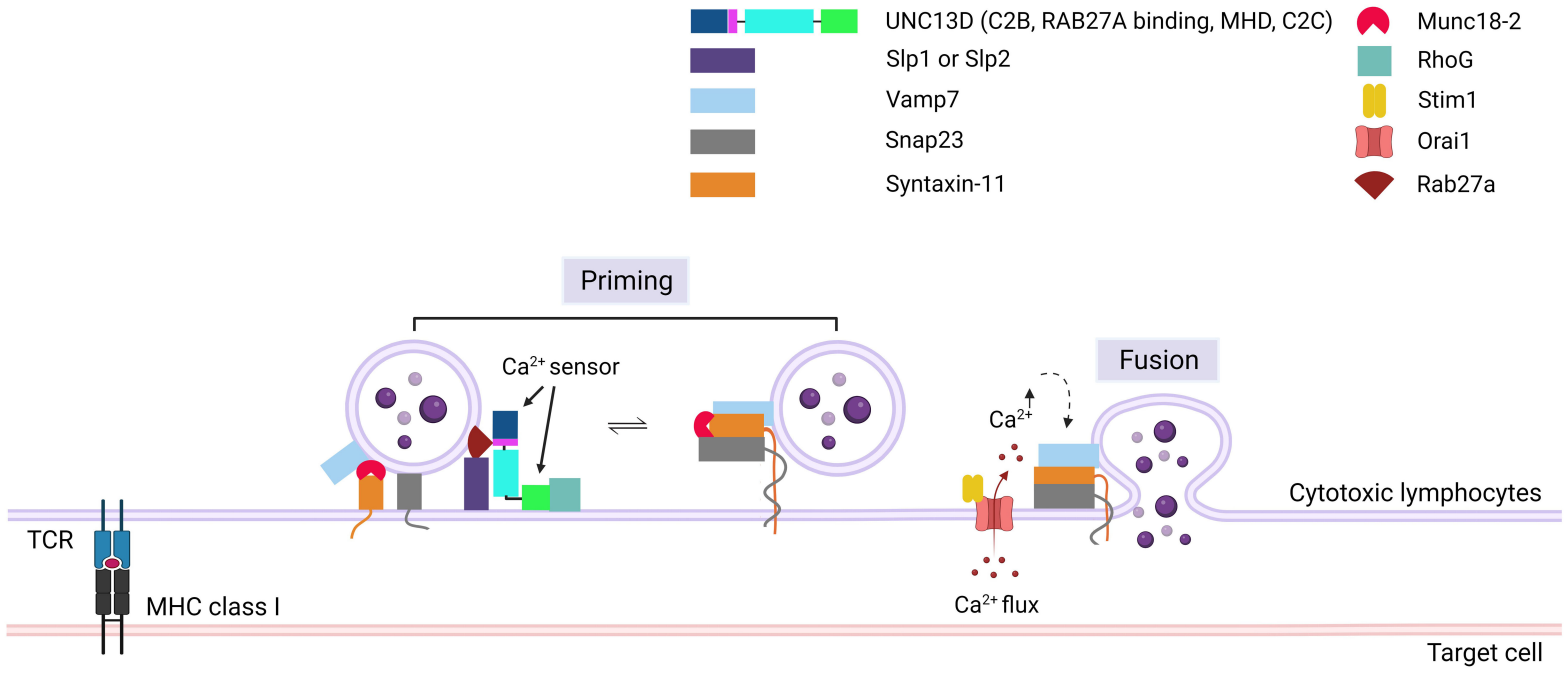
Role of UNC13D in cytotoxic granule priming. The formation of trans-SNARE complex is presented. Docking of mature cytotoxic granules at the PM is achieved through the interaction of Rab27a with Slp1 or Slp2. Moreover, cytotoxic granules interact with a docking complex formed by Munc18-2 and Stx11 in the closed conformation. The docked granules are primed by UNC13D following calcium flux, possibly through the UNC13D-mediated conversion of syntaxin-11 to an open conformation by displacing Munc18-2. Additionally, UNC13D is involved in the formation of an initial bridge connecting the granule membrane to the plasma membrane by interacting with RhoG via its C2C domain. Finally, a complex known as SNARE is established by the interaction of plasma membrane Snap23 and syntaxin-11 with granule membrane Vamp7, triggering the fusion of vesicles with the plasma membrane, resulting in the release of granule content into the synaptic cleft at the immunological synapse. Orai1, Calcium release-activated calcium channel protein 1; Slp1/2, synaptotagmin-like proteins1/2; SNARE, soluble N-ethylmaleimide-sensitive factor accessory protein receptor; RhoG, Ras Homolog Family Member G; Stim1, Stromal interaction molecule 1; Snap23, Synaptosomal-associated protein 23; Vamp7, Vesicle-associated membrane protein 7.

Following the tethering process, SNARE proteins play critical roles in the tight docking of cytotoxic granules to the PM. The priming event involves the formation of a substantial trans-protein complex consisting of SNARE proteins on vesicles (v-SNAREs) and on the PM (t-SNAREs) (**Figure 4**) (48). The UNC13 family members play a role in priming synaptic vesicles in presynaptic neurons (10, 49), whereas UNC13D has a similar function in CTLs and NK cells (4). Experiments with v- and t-SNARE liposomes revealed that UNC13D facilitates the formation of trans-SNARE complexes and that the fusion of liposomes is Ca^2+^-dependent. Boswell et al. reported that the C2B domain of UNC13D is crucial for Ca^2+^-dependent binding to t-SNAREs, whereas the C2C domain is responsible for Ca^2+^-dependent interactions with phospholipids (47). At the immunological synapse, the interactions between t-SNARE proteins (SNAP23 and STX11) and v-SNARE proteins (VAMP7) can cause the trans-core SNARE complex to overcome the barrier of negative repulsion between two lipid membranes and water molecules. Additionally, the MHD domains of the UNC13 family proteins are associated with priming functions (50). The phenotype observed in patients with FHL3 also supports the role of UNC13D in this step. Mutations in UNC13D result in the formation of cells in which granules can dock at the PM but are unable to release their contents, which indicates that UNC13D plays a vital role in priming cytotoxic lytic granules before fusion can occur (6, 20).

During the formation of the trans-SNARE complex, the inhibition of STX11 expression by MUNC18-2 may be released by UNC13D (51). MUNC18-2 (STXBP2) maintains an unsuitable (closed) state for STX to form the trans-SNARE complex. The interaction of UNC13D with MUNC18-2 allows the transition of STX11 to an open state, facilitating the formation of the trans-SNARE complex. This interaction is consistent with the finding that the MHD domains of UNC13A interact with STX1/STXBP1 in neurons, enabling STXBP1 to recruit VAMP2 (52–55). In addition, MUNC18-2 has been co-immunoprecipitated with UNC13D in platelets (56).

### 3.4 Role of UNC13D in the endoplasmic reticulum

The cyclic guanosine monophosphate (GMP)-adenosine monophosphate (cGAMP) synthase (cGAS)-stimulator of interferon genes (STING) pathway is associated with aberrant exogenously or endogenously derived cytosolic DNA (57). Binding of cGAS to aberrant DNA results in the generation of cGAMP and activation of STING, which is located in the ER. During STING activation, the STING dimer is oligomerized and transported to the Golgi apparatus via the ER-Golgi intermediate compartment (ERGIC). Finally, the IRF3 and NF-κB pathways are activated to promote inflammatory reactions. UNC13D inhibits STING signaling by attenuating its oligomerization in the ER (34). A recent study showed that UNC13D co-precipitates with STING and co-localizes in the ER. Overexpression of UNC13D in HeLa cells inhibits the translocation of STING to ERGIC (34). Accordingly, patients with FHL3 having low UNC13D levels show hyperinflammatory responses (6).

## 4 Genetic characteristics of UNC13D defects

UNC13D is composed of 32 exons and 31 introns (6). To date, at least 200 distinct mutations in the UNC13D gene have been documented as a contributing factor to FHL3, in which approximately 36.6%, 15.6% and 47.6% exhibited homozygous, heterozygous, compound heterozygous mutations, respectively (58). A systemic study of 322 patients showed that mutations were identified in 53.4% of exons and 46.6% of introns. Patients with severe features demonstrated a 1.6-fold increase in the prevalence of exonic mutations compared to intronic mutations, whereas the distribution of exonic and intronic mutations was nearly balanced in patients with mild features (58). Missense and splice mutations were the most frequent in severe and mild feature patients, respectively.

Among intronic mutations, the variant c.118-308C>T and the 253-kb inversion along with c.117 + 143A>G, c.118-307G>A, c.754-1G>C, c.1596 + 1G>C are notable pathogenic variants of UNC13D (59–64) (**Table 2**). Among exonic mutations, at least six pathogenic mutations were identified by cell-based functional assays (**Table 2**). Three of them (E379K, L403P, R414C) were located in the RAB27A interaction domain, one (G863D) in the MHD2 domain, one (A918) in the C2C domain (**Figure 1**), one (T76M) in the N-terminal.

**Table 2:** Notable pathogenic mutations of UNC13D .

Site	Variant	Features	Demographic Distribution	References
Intron	c.118-308C>T	Disruption of lymphocyte-specific enhancer disrupts an ELF1 transcription factor binding site and has a negative regulatory effect on transcription.	Northern - European Korean Japan North America	(60, 62, 63)
	(253-kb inversion)	This affects the 3’- end of the transcript and consequently abolishes the expression of the UNC13D protein.	Scandinavia North America	(60, 63)
	c.1389 + 1G>A	This sequence change affects a donor splice site in intron 15 of the UNC13D gene. It is expected to disrupt RNA splicing. Variants that disrupt the donor or acceptor splice site typically lead to a loss of protein function.	European-American	(6, 41, 65–69)
	c.1848 + 1G>C	This sequence change affects a donor splice site in intron 20 of the UNC13D gene. It is expected to disrupt RNA splicing.	ND	(63)
	c.2447 + 1G>A	This sequence change affects a donor splice site in intron 25 of the UNC13D gene. It is expected to disrupt RNA splicing and likely results in an absent or disrupted protein product.	ND	(69)
	c.753 + 1G>T	Canonical splice site variant in a gene for which loss-of-function is a known mechanism of disease. Studies have shown that disruption of this splice site results in skipping of exon 9 and introduces a premature termination codon.	Caucasian	(6, 41, 66, 67, 69–75)
	c.754-1G>C	This sequence change affects an acceptor splice site in intron 9 of the UNC13D gene. It is expected to disrupt RNA splicing.	Korean Japan	(58, 62)
	c.1596 + 1G>C	This sequence change affects a donor splice site in intron 18 of the UNC13D gene. It is expected to disrupt RNA splicing.	Japan	(59)
	c.117 + 143A>G	Disrupts an enhancer and has been associated with reduced gene transcription and macrophage activation.	ND	(64)
	c.118-307G>A	Disruption of lymphocyte-specific enhancer.	China	(61, 63)
Exon	c.2588G>A (p.G863D)	Results in a non-conservative amino acid change located in the MUN domain of the encoded protein sequence.	East Asian	(76)
	c.1240C>T (p.R414C)	This variant resulting in a non-conservative amino acid change in the encoded protein sequence. The missense change has been observed in individuals with atypical hemophagocytic lymphohistiocytosis-like phenotypes.	Caucasian	(77–81)
	c.2346_2349del (p.R782fs)	Frameshift variant predicted to result in protein truncation or nonsense mediated decay in a gene for which loss-of-function is a known mechanism of disease	Caucasian	(69, 70, 82–87)
	c.640C>T (p.R214X)	This sequence change creates a premature translational stop signal (p.Arg214*) in the UNC13D gene. It is expected to result in an absent or disrupted protein product.	Japan China Turkey	(59, 83, 88)
	c.2695C>T (p.R899X)	The mutation in exon 28 of UNC13D causes a premature stop codon and encodes munc13-4(1–899), which lacks the C-terminal C2 domain.	African American	(69)
	c.551G>A (p.W184X)	This variant creates a premature translational stop signal in the UNC13D gene. It is expected to result in an absent or disrupted protein product.	European-American	(69)
	c.766C>T (p.R256X)	This variant is a stop-gained variant that is predicted to result in loss of normal protein function either through protein truncation or nonsense-mediated mRNA decay.	US- Caucasian	(58, 69)
	c.1847A>G (p.E616G)	This variant results in a non-conservative amino acid change located in the Munc13 homology 1 domain (IPR014770) of the encoded protein sequence.	Caucasian	(58)
	c.1208T>C (p.L403P)	Null compared with WT activity in the functional assay	Turkish	(82)
	c.2588G>A (p.G863D)	22% of WT activity in the functional assay	East Asian	(76)
	c.2753C>A (p.A918D)	17% of WT activity in the functional assay	Caucasian	(79)
	c.1240C>T (p.R414C)	20% of WT activity in the functional assay	Caucasian	(79)
	c.1135G>A (p.E379K)	15% of WT activity in the functional assay	South Asian	(89)
	c.227C>T (p.T76M)	22% of WT activity in the functional assay	African American	(89)

ND, No data.

It is important to explore how the expression of UNC13D is regulated in different lymphocyte subsets, given the essential role this protein plays in lymphocyte cytotoxicity (6). In 2000, Koch and his team reported a ubiquitous UNC13D expression pattern; however, a more in-depth analysis has not been performed since that initial finding (9). Remarkably, mutations occurring in an evolutionarily conserved region of UNC13D intron 1 have been frequently associated with the development of FHL3 (60–63). This sequence serves as an overall enhancer and regulates the expression of an alternative UNC13D isoform that has a unique N-terminus (60, 90). Using isoform specific antibodies, Galgano et al. (91) revealed that the alternative UNC13D isoform, which possesses a unique N-terminus, is preferentially expressed in human lymphocytes and platelets, whereas the conventional isoform is mainly expressed in monocytes and neutrophils. The distinct N-terminal regions of the two isoforms did not alter the localization or trafficking of UNC13D to the immunological synapse in cytotoxic T cells. Additionally, the ectopic expression of both isoforms proficiently restored exocytosis in T cells lacking UNC13D, which were derived from patients with FHL3. The research established that the conventional and alternative isoforms of UNC13D show different expression profiles in hematopoietic cell subsets, while exhibiting similar localization and contribution in T cell exocytosis (91). Although similar localization and functional contribution of both N-terminals, there might be some difference in susceptibility to bacterial infection and clinical manifestations in splice 1 mutation patients. In FHL3 patients, c.118-308C>T intronic homozygous mutation reduced conventional and alternative UNC13D transcript expression and affected cytotoxic exocytosis (90). However, in recurrent macrophage activation syndrome (MAS) and systemic juvenile idiopathic arthritis patients, c.117 + 143A>G mutation did not affect UNC13D expression in other immune cell types (64).

## 5 Pathophysiological roles of UNC13D in immune diseases

### 5.1 FHL3

Hemophagocytic lymphohistiocytosis (HLH) is a life-threatening hyperinflammatory syndrome caused by the defective functions of CTLs, NK cells, and macrophages, resulting in cytokine storms and immune cell-mediated damage across multiple organ systems (92, 93). Dysregulated mechanisms responsible for impaired granule-mediated cytotoxicity result in prolonged contact between lymphocytes and their target cells. In addition, the inability of the immune system to eliminate the target cells, antigen-presenting cells, and cytotoxic cells results in failure to terminate the immune response (93–95). Consequently, a continuous increase is observed in cytokine levels, T-cell proliferation, and macrophage activation, ultimately leading to multi-organ failure (96, 97). HLH is typically divided into primary and secondary forms, based on the presence or absence of genetic defects. Familial HLH (FHL), a term for primary HLH, is inherited as an autosomal recessive condition, whereas secondary HLH is caused by infections, autoimmune disease, or malignancies (98, 99).

Cytotoxicity is a complex process that involves the generation of lytic granules containing perforin and granzyme B. These granules are then transported and docked to the membrane, followed by exocytosis at the immunological synaptic interphase. Ultimately, this process leads to target cell lysis (4). To date, four genetic abnormalities—PRF1 encoding perforin (FHL2) (100), UNC13D encoding Munc13-4 (FHL3) (6), STX11 encoding syntaxin-11 (FHL4) (101), and STXBP2 encoding Munc18-2 (FHL5)—have been detected in FHL (102, 103). Perforin acts as a cytolytic effector molecule for CTLs and NK cells, creating pore-like structures in the membranes of target cells. In contrast, UNC13D, syntaxin-11, and Munc18–2 play roles in the intracellular trafficking and membrane fusion of cytolytic granules. Thus, the cytolytic activity of NK cells and CTLs is inherently impaired in patients with FHL (104). UNC13D deficiency was initially reported in 2003 and is the second most prevalent genetically determined variant of FHL, referred to as FHL3. It accounts for 20–40% of the FHL incidences, depending on geographic distribution and ethnicity (85, 105). More than 100 mutations have been identified in the *UNC13D* gene, distributed across 32 exons, and the most frequent molecular alterations are those affecting mRNA splicing (71, 72, 83).

### 5.2 Macrophage activation syndrome

MAS is a type of HLH associated with rheumatological diseases such as systemic juvenile idiopathic arthritis (sJIA) (106). The clinical and laboratory characteristics of MAS and HLH exhibit striking similarities (107). In the last few years, an association has been observed between MAS in patients with juvenile idiopathic arthritis (JIA) and UNC13D mutations, particularly polymorphic variants in critical regulatory regions (64, 108, 109). Specifically, a novel functional intronic alteration within UNC13D, c.117 + 143A>G, was found to be related to MAS and JIA (64). Yang et al. identified a homozygous missense mutation in UNC13D (c.2588G>A, p. G863D), a hotspot mutation in the Chinese population, associated with recurrent MAS (110). This relationship may partially explain the increased risk of MAS development in specific patients. Studies on patients with *UNC13D* mutations experiencing juvenile polymyositis and MAS provide important insights into the pathogenesis of familial HLH (110).

### 5.3 JIA

JIA is the most common pediatric rheumatological disease (111) and is classified into the following subtypes: oligoarticular, polyarticular, enthesitis-related, psoriatic, and systemic JIA. HLH is the most prevalent in patients with sJIA, who are at the risk of MAS development (112). Several research groups have consistently reported a decrease in perforin levels in both CTLs and NK cells, along with impaired NK cell function in patients with sJIA (113, 114). Hazen et al. reported that a child suffering from JIA had compound heterozygous mutations in UNC13D; one allele contained a known mutation in the splice donor site of intron 9 (753 + 3 [G>A]), whereas the other allele carried a novel missense mutation in exon 18 (1579 [C>T] R527W), which reduced the cytotoxic function of NK cells. These findings suggest that sJIA may be considered a form of haemophagocytic syndrome (115). Following this, variations in UNC13D were observed in three of eighteen individuals with s-JIA/MAS, two of whom had biallelic mutations (109). Recently, exome sequencing analysis revealed two heterozygous UNC13D variants (p.R727Q and p.A59T) in an individual diagnosed with JIA (116).

### 5.4 Autoimmune lymphoproliferative syndrome

ALPS is a well-known disease resulting from genetic defects in components of the FAS pathway, including FAS/TNFRSF6, FASL/TNFSF6, and CASP10 (58). Sequencing analysis identified four rare missense variants of UNC13D, including p.Cys112Ser, p.Val781Ile, and a haplotype containing both p.Ile848Leu and p.Ala995Pro, in three patients with ALPS. Additionally, a homozygous missense variation (c.2588G > A, p.G863D) was found in the *UNC13D* gene by Gu et al. (117). Such variations ultimately lead to excessive activation of T cells and macrophages, one of which is associated with the impaired development of Treg cells and severe ALPS-like symptoms (117). PRF1 and UNC13D can serve as risk factors for the development of ALPS in patients who do not exhibit any defects in the FAS pathway (118, 119). These findings suggest that rare loss-of-function variations in UND13D are potential risk factors for ALPS development (120).

## 6 Techniques for evaluating UNC13D defects

For patients who are suspected to have FHL3, initial step involves the detection of its protein and an assessment of its functional properties. Although western blotting was a prevalent method for detection; however, it is important to note that SDS-PAGE can alter the secondary structure of proteins, which could lead to variations in the detection results. It has been documented that the expression of UNC13D is markedly greater in platelets than in peripheral blood mononuclear cells (PBMCs) and in the populations of CD8, CD4, CD14, and CD19 (121). The detection of UNC13D protein in platelets using western blotting or flow cytometry is an effective method for screening individuals diagnosed with FHL3. However, in the acute phase of disease that required platelet transfusion, it was challenging to identify FHL3 using western blot analysis. Alternatively, the recently introduced flow cytometric analysis of intraplatelet UNC13D protein expression has revealed the existence of bimodal populations of platelets, which include both normal and those lacking UNC13D (121). Although the flow cytometry tends to be more sensitive, but the results may vary depending on the antibody selection (122).

To provide a comprehensive genetic evaluation of FHL3 patients, sequence analysis can be chosen either by direct Sanger sequencing or by Next-Generation Sequencing (123, 124). However, the evaluation of pathogenicity of each genetic variant is required. FHL3 exhibited a range of clinical manifestations that were both diverse and complex. In some cases, patients showed atypical symptoms, resulting in challenges in achieving an accurate diagnosis. Zhao et al. suggests that the expression of UNC13D protein may not fully represent the pathogenic implications of UNC13D variants (122).

Recently *in vitro* cell-based functional assays for the evaluation of UNC13D variants were introduced because western blotting, flow cytometry and sequencing techniques could not directly indicate the functional status of UNC13D and immunotherapies can distort the cytotoxicity assay (89). In this assay, after knocking out endogenous UNC13D, its variant was introduced into cells and cytotoxic activity of CD8+ T and NK cells and their degranulation capacity were measured. These functional assays can distinguish pathogenic from nonpathogenic mutations. Interestingly, late-onset manifestation of mutation was also functionally evaluated.

## 7 Therapeutic approach in human UNC13D defects

Patients carrying mutations in the UNC13D gene, whether homozygous or compound heterozygous, that result in abnormal, truncated, or missing UNC13D variants, suffer from familial hemophagocytic lymphohistiocytosis type 3. Individuals diagnosed with FHL3 often develop acute HLH within their first year of life, and commonly manifest with fever, hepatosplenomegaly, cytopenia, and distinctive laboratory features, frequently presenting with neurological manifestations (125–127). So, the first phase of treatment focuses on managing hyper-inflammation and etoposide-based chemotherapy is administered. Alternatively, immunotherapy utilizing antithymocyte globulin (128–131) is employed as a first-line therapy, although more favorable outcomes have been observed with the monoclonal anti-CD52 antibody alemtuzumab (132–135). Combinatorial immunochemotherapy is employed to regulate the cytokine storm and inhibit cellular proliferation; however, it frequently leads to significant side effects after prolonged treatment (92).

**Figure 5:**
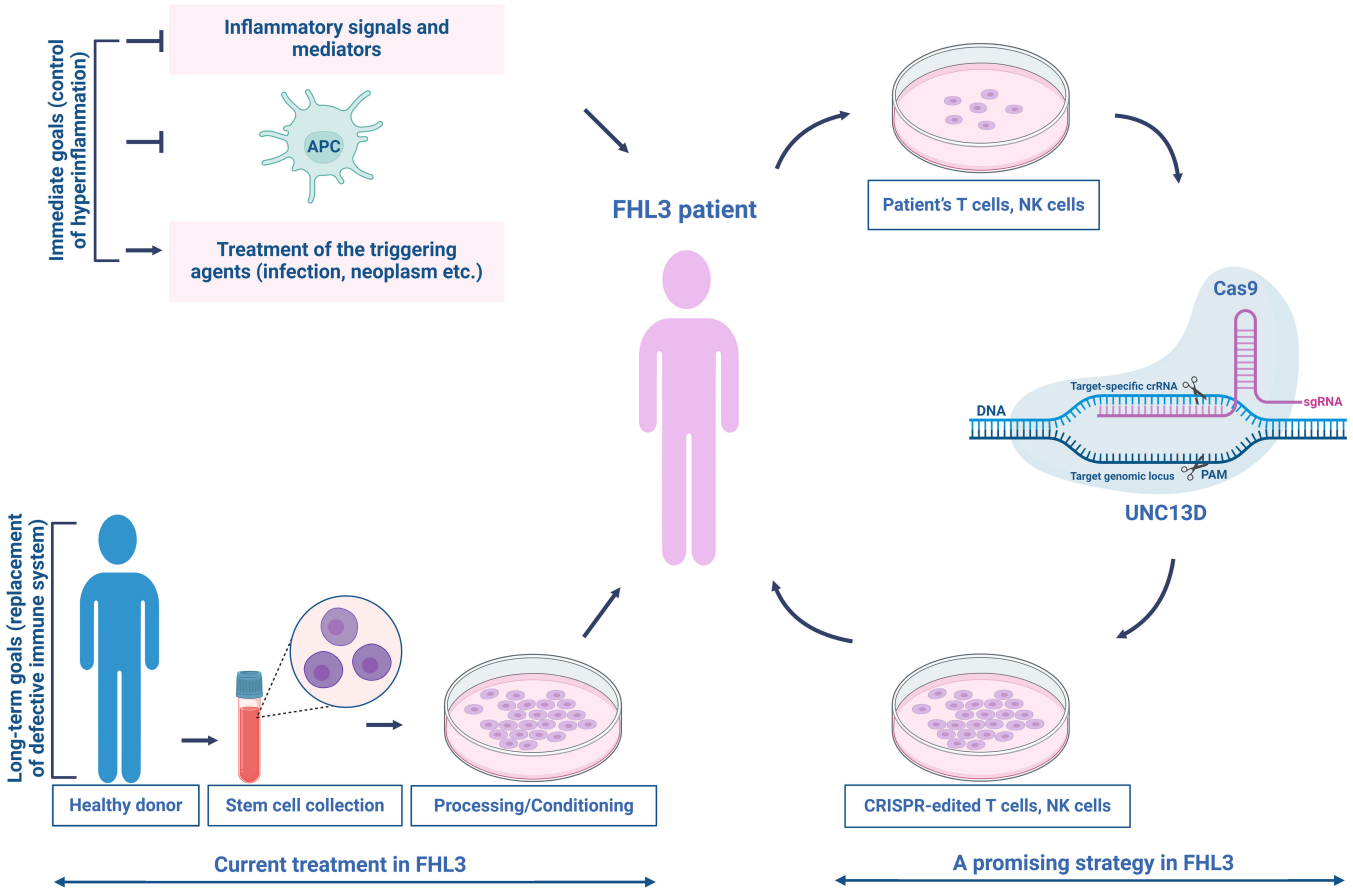
Current treatment and a promising therapeutic strategy for patients with FHL3. The current treatment for FHL3 is typically divided into two phases—controlling hyperinflammation and replacing the defective immune system. In the first phase, the standard of care consists of etoposide-based chemotherapy (HLH-94 treatment protocol) to control severe inflammation, eradicate over-stimulated antigen-presenting cells, and manage the triggering agent (infection, neoplasm, etc.). The second phase commonly involves HSC transplantation after myeloablative/reductive conditioning. Targeted genome editing could offer a solution to the problems associated with current treatments, restoring NK/T cell cytotoxic function. This promising strategy establishes a beginning for the clinical integration of autologous NK/T cell therapy in patients with FHL3.

For the replacement of dysfunctional immune system (**Figure 5**), the second phase of treatment usually involves hematopoietic stem cell (HSC) transplantation after myeloablative/reductive conditioning; however, the mortality rate remains high (92, 136–139). The incomplete remission of disease activity, conditioning-related toxicities, and graft rejection during the transplantation are major risks that pose significant challenges (140, 141). The long-term survival rate of patients with FHL is approximately 50% (137, 142), making it imperative for clinical advancements using new methods to improve treatment outcomes and reduce side effects.

Recently, gene therapy is extensively studied for the functional recovery for the UNC13D defects. As the second most common type of primary HLH, FHL3 is a potential candidate for gene therapy. A feasible strategy is to employ autologous, repaired T cells as a transitional step toward transplant therapy to enhance the health of the patient and minimize the potential risks associated with HSC transplantation (HSCT). The first gene therapy study on FHL3 was published by Soheili et al. (143), which involved the transduction of activated peripheral blood mononuclear cells from three FHL3 patients. This was achieved using either a vesicular stomatitis virus G protein or a measles H/F pseudotyped LV vector encoding a complementary DNA product from human *UNC13D*. Transplanting EBV-induced tumors into NSG mice, followed by the infusion of transduced T cells from patient with FHL3, resulted in a noticeable reduction in tumor size (143). Soheili et al. successfully transduced HSC derived from UNC13D null (Jinx) mice, a model for FHL3, demonstrating that gene therapy improved the *in vitro* metrics and viral clearance (144). In 2019, Dettmer et al. used a gamma retrovirus-based gene therapy construct to transduce cells from patient with FHL3 cells, resulting in improved degranulation capacity and cytotoxicity in transduced cells (145). Previous research showed that introducing the *UNC13D* gene into Jinx HSCs using lentiviral transfer successfully restored T-cell function in the FHL3 (Jinx) disease model, with 15% of degranulation-competent cells being sufficient to trigger a degranulation response comparable to that of the wild type (146). Despite the advancements in lentiviral and retroviral gene therapy, concerns still remain regarding insertional mutagenesis (147–149) and the regulation of UNC13D expression (9, 30, 150, 151).

Recent advancements in targeted genome editing have shown great promise in addressing these challenges. Dettmer et al. (145) employed a CRISPR/Cas9-based strategy to delete an intronic cryptic splice donor site within UNC13D, achieving a high success rate (**Figure 5**). Jinx mice receiving UNC13D-edited HSCs exhibited notable chimerism without any detectable off-target effects. These mice were completely protected against lymphocytic choriomeningitis virus-induced HLH and showed no signs of clonal T-cell expansion or leukemogenesis (152). Moreover, repaired long-lived T memory stem (T_SCM_) cells can provide extended protection, as genetically modified T cells can survive for over ten years (153, 154). T_SCM_ cells, similar to naïve T (T_N_) cells, have a low differentiation ability compared to other memory T-cell subsets and are suggested to occupy the highest position in the T-cell hierarchy within a progressive model of T-cell differentiation (155). Following allogeneic HSCT, only T_N_ and T_SCM_ cells can regenerate the entire spectrum of T-memory cell subsets, including T_SCM_ cells (156). In FHL, many children have genetic loss-of-function mutations that disrupt the functions of proteins necessary for the normal cytotoxic activities of T and NK cells (93). Recently, the efficacy of precisely repaired autologous T cells in addressing FHL has been highlighted. Adeno-associated virus-templated CRISPR-Cas9 was employed to successfully restore the function of memory T cells lacking perforin-1 in mice. These repaired cells were subsequently used to treat life-threatening hyperinflammation induced by EBV in a mouse model of FHL. Additionally, T cells derived from pediatric patients with FHL2 and FHL3 were effectively expanded and repaired, resulting in the recovery of CD8 + T-cell cytotoxicity while maintaining a T_SCM_ cell-like phenotype (157).

Autologous gene-modified T cells have been utilized in cancer treatment of numerous patients, demonstrating their safety and lack of malignant transformation. Accordingly, the potential impact of unwanted on-and off-target events may be less critical for autologous T cells (157, 158). However, to apply precise gene editing in clinical practice, a comprehensive evaluation of the potential off-target sites is required (**Figure 5**).

## 8 Discussion and future direction

Although notable progress has been made in understanding the role of UNC13D in the nervous system, its function in the immune system remains poorly characterized. Recent cancer studies have highlighted its importance (12, 13). The regulation of vesicle trafficking and its contribution to tumor progression need further exploration. In the immune system, the role of UNC13D in the priming of cytotoxic granules is relatively well established. However, its involvement in other steps, such as the biogenesis and tethering of cytotoxic granules, is not well understood. In particular, the association of its biological role in the immune system with the progression of immunological diseases needs to be characterized. For therapeutic development involving UNC13D, lipid nanoparticles for the delivery of therapeutic genes must be examined because of their potential in gene therapy (159, 160).
